# Klippel-Trenaunay Syndrome: A Case Study of Severe Anemia in a Rare Vascular Disorder

**DOI:** 10.7759/cureus.75725

**Published:** 2024-12-15

**Authors:** Usamah Al-Anbagi, Ibrahim Elmakaty, Hadeel M Al-Zoubi, Abdulqadir J Nashwan, Muhammad Sharif

**Affiliations:** 1 Internal Medicine Department, Hamad Medical Corporation, Doha, QAT; 2 Nursing and Midwifery Research Department, Hamad Medical Corporation, Doha, QAT

**Keywords:** computed tomography (ct scan), hypertrophy, klippel-trenaunay syndrome (kts), magnetic resonance imaging (mri), varicosities, vascular malformations

## Abstract

Klippel-Trenaunay syndrome (KTS) is a rare congenital vascular disorder involving varicosities, cutaneous vascular malformations, and hypertrophy of soft tissues and bones. It is often linked to *PIK3CA* gene mutations. It affects the lymphatic, capillary, and venous systems. The diagnosis is usually based on clinical presentation, supplemented by magnetic resonance imaging (MRI) and computed tomography (CT) imaging. This case involves a 43-year-old male diagnosed with KTS after presenting with severe anemia (hemoglobin 2.5 g/dL) and left lower limb swelling with varicosities. Investigations revealed hepatosplenomegaly, hemangiomas, rectosigmoid malformations, an enlarged inferior vena cava (IVC), and vascular congestion. MRI confirmed an extensive veno-lymphatic malformation in the left lower limb. The case highlights KTS's complex presentation, emphasizing the importance of timely diagnosis, multidisciplinary care, and ongoing monitoring to manage its complications. Further research is needed to enhance treatment strategies for this rare condition.

## Introduction

Klippel-Trenaunay syndrome (KTS) is a rare congenital disorder characterized by a triad of clinical features: (1) capillary malformations, (2) venous malformations or varicosities, and (3) hypertrophy of the affected tissues [1-4]. According to the 2018 classification by the International Society for the Study of Vascular Anomalies, KTS can occur with or without associated lymphatic malformations [2]. While predominantly sporadic, familial cases have been documented, suggesting a complex genetic basis [5,6]. Most patients exhibit postzygotic somatic variants in the phosphatidylinositol-4,5-bisphosphate 3-kinase catalytic subunit alpha (*PIK3CA*) gene, contributing to the disorder's pathophysiology through mechanisms that drive angiogenesis and tissue overgrowth [7,8].

KTS often presents with varying degrees of severity, influenced by the timing of vascular development in utero and the specific gene variants involved. Histopathological examination reveals a range of vascular malformations, including ectatic capillary vessels, irregular venous structures, and lymphatic anomalies [9,10].

Management of KTS requires a multidisciplinary approach at specialized centers involving various medical specialties. Treatment plans are individualized, focusing on supportive care to manage complications. Further medical or surgical interventions may be needed in cases with significant symptoms. A key advancement is the FDA approval of alpelisib as the first targeted therapy for *PIK3CA*-related overgrowth spectrum (PROS), improving treatment options for KTS patients [11].

Given its complexity, KTS often requires ongoing monitoring and tailored care plans to address the diverse needs of affected individuals. This case report aims to contribute to understanding KTS by discussing a patient who presented with significant systemic symptoms, emphasizing the importance of comprehensive evaluation and management strategies.

## Case presentation

A 43-year-old man presented to the emergency department with a one-week history of generalized weakness, fatigue, shortness of breath, and dizziness. He also reported intermittent rectal bleeding for the past seven days, occurring two to three times per day with bowel movements without associated diarrhea or constipation. Ten years prior, he had a similar episode of rectal bleeding that was diagnosed as hemorrhoids and treated with hemorrhoidectomy. The patient denied any history of hematemesis, smoking, or alcohol use but mentioned a prior left ankle surgery for a suspected tumor.

**Figure 1:**
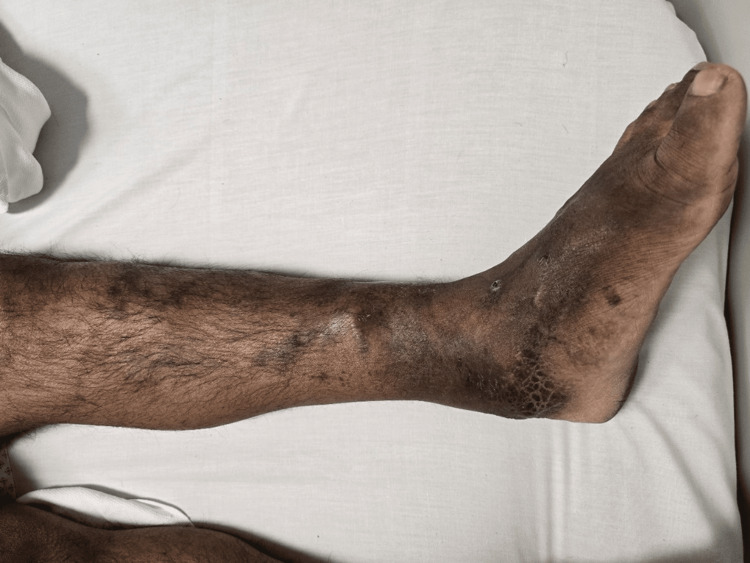
The left lower limb shows a swollen leg along with superficial varicosities.

On examination, he was pale with a temperature of 36.5°C, a heart rate of 103 bpm, a respiratory rate of 18 breaths/minute, a blood pressure of 103/62 mmHg, and an oxygen saturation of 100% on room air. His conjunctiva and skin were pale, and he had a pterygium on his right eye and an erythematous rash across the bridge of his nose and cheeks. Cardiovascular examination revealed a 2/6 systolic murmur at the apex, while the respiratory, neurological, and abdominal exams were normal. Examination of the lower limbs showed left leg swelling, pitting edema, hyperpigmentation, dilated veins, and multiple lichenified plaques with dry, scaling skin (Figure 1).

The initial laboratory workup revealed severe microcytic hypochromic anemia (hemoglobin 2.7 g/dL) and mild liver function abnormalities (Table 1).

**Table 1:** Laboratory investigations WBC/total leukocytes: white blood cells; MCV: mean corpuscular volume; MCH: mean corpuscular hemoglobin; BUN/urea: blood urea nitrogen (reported as serum urea); HbA1c: glycated hemoglobin; AST: aspartate transaminase; ALT: alanine transaminase

Parameters	On Admission	3rd Day	On Discharge	Reference Values
Total leukocytes	4	5.3	5	(6.2x10^3^/uL)
Hematocrit	10.7	30.7	32.9	(40-50%)
Hemoglobin (g/dL)	2.5	9.3	9.7	(13-17 g/dL)
MCV (fL)	65.6	77.7	79.9	(83-101 fL)
MCH (pg)	15.3	23.5	23.5	(27-32 pg)
Platelet (×10^3^/uL)	348	255	237	(150-410x10^3^/uL)
Serum potassium K (mmol/L)	4.3	4	3.7	(3.5-5.3)
Serum sodium (mmol/L)	139	143	140	(133-146)
Serum calcium (mmol/L)	2.14	-	-	(2.2-2.6)
Serum urea (mmol/L)	1.7	1.9	1.3	(2.5-7.8)
Serum creatinine (µmol/L)	70	72	69	(62-106)
HbA1c	5.8	-	-	<6%
Serum albumin (g/L)	36	29	31	(35-50)
Serum total protein (g/L)	76	64	70	(60-80)
AST (IU/L)	421	191	94	(0-41)
ALT (IU/L)	134	38	8	(0-41)
Alkaline phosphatase (U/L)	202	149	137	(40-129)
Serum chloride (mmol/L)	109	109	106	(95-108)
Serum bicarbonate (mmol/L)	19	22	23	(22-29)

**Figure 2:**
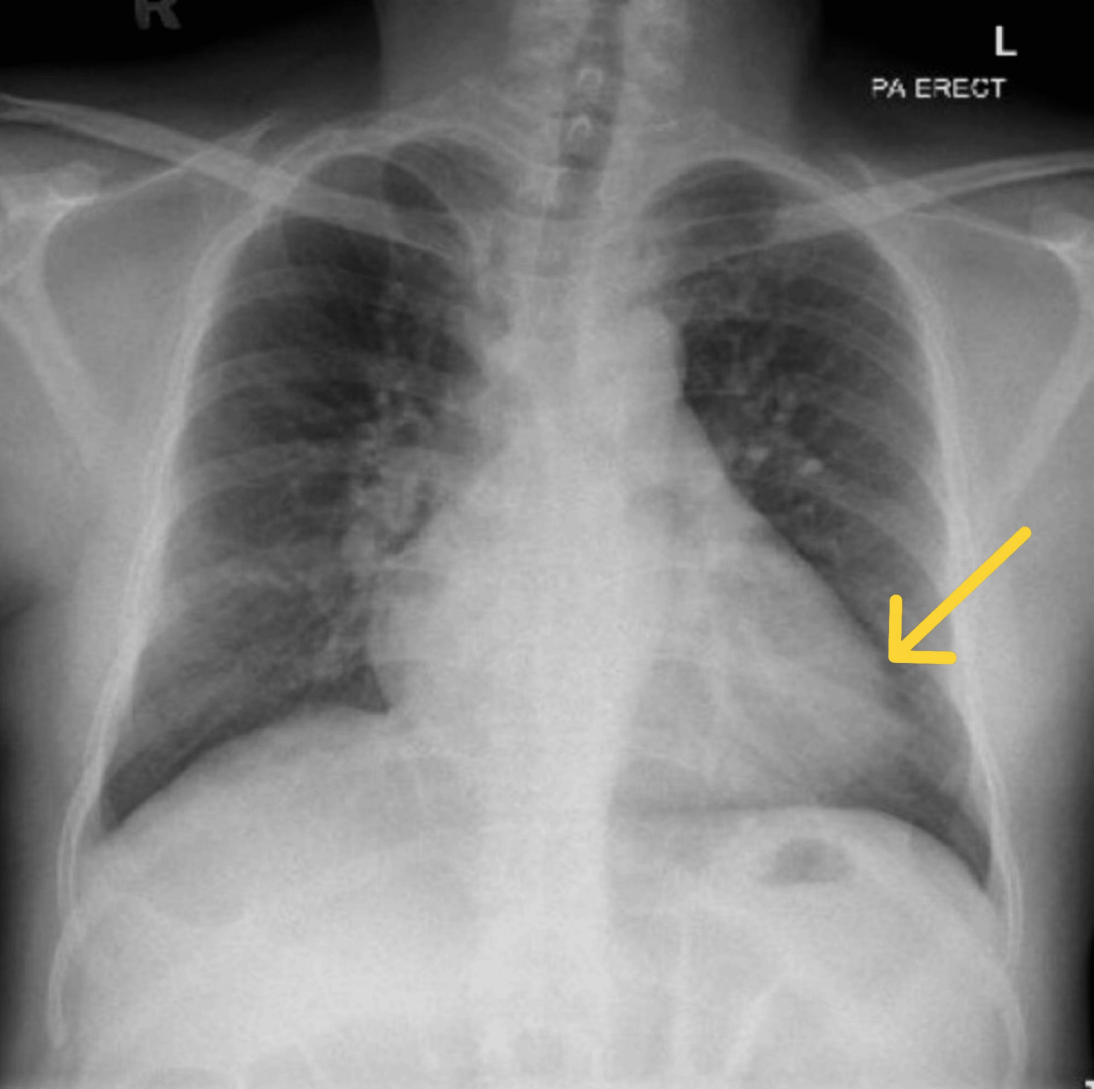
A chest X-ray posteroanterior view revealed mild cardiomegaly.

A chest X-ray showed cardiomegaly (Figure 2), while an electrocardiogram was normal. The gastrointestinal bleeding protocol was initiated with proton pump inhibitors and blood transfusions. The patient was admitted for stabilization with a target hemoglobin level above 8 g/dL.

**Figure 3:**
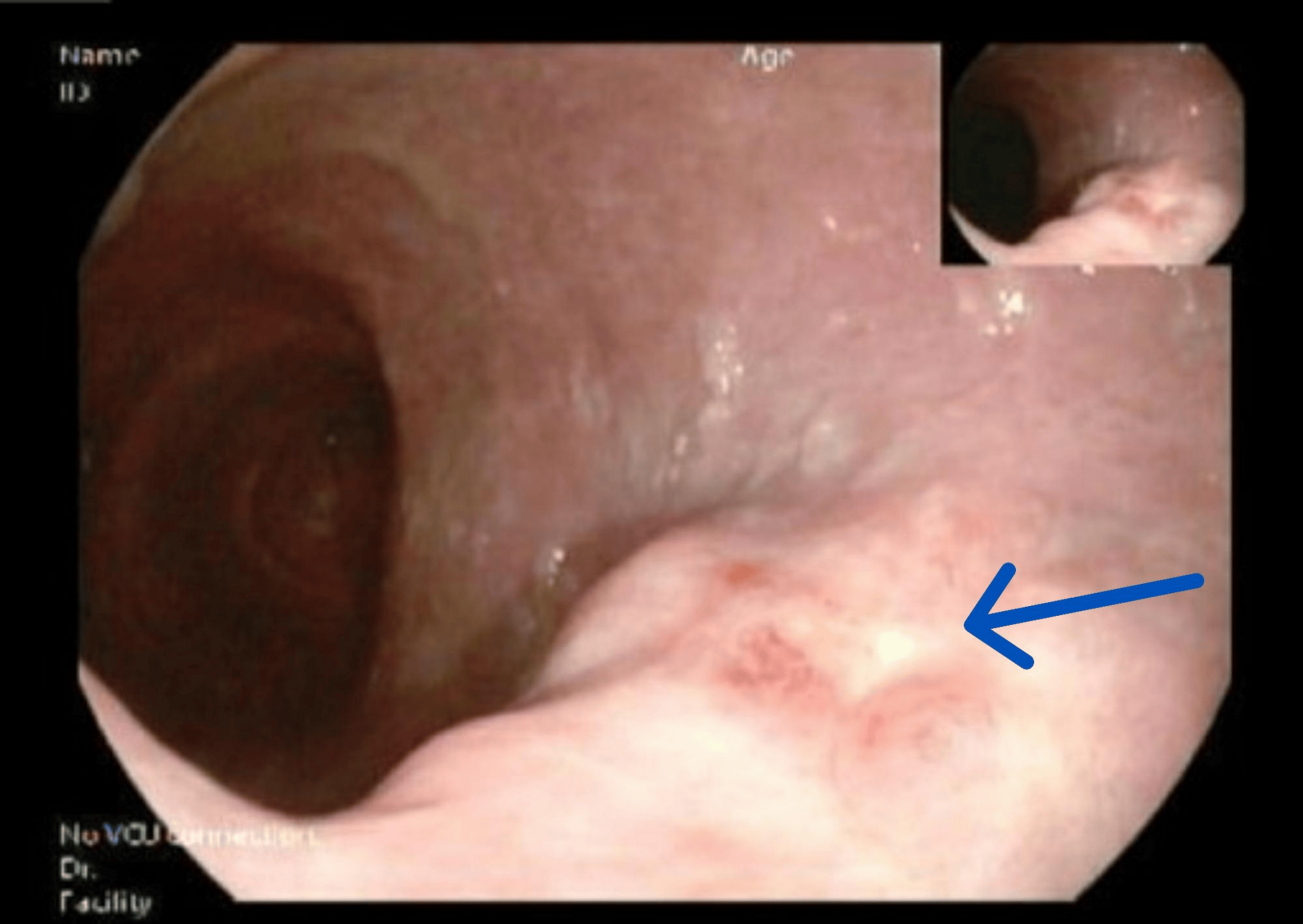
Colonoscopy revealed a rectosigmoid ulcer.

Echocardiography on the second day showed mildly reduced left ventricular function (LVEF 50%), diastolic dysfunction, and a dilated left atrium. Abdominal ultrasound revealed hepatic steatosis and splenomegaly with hyperechoic lesions. Endoscopic evaluations identified gastritis, rectosigmoid ulcers (Figure 3), venous congestion, and large internal hemorrhoids.

**Figure 4:**
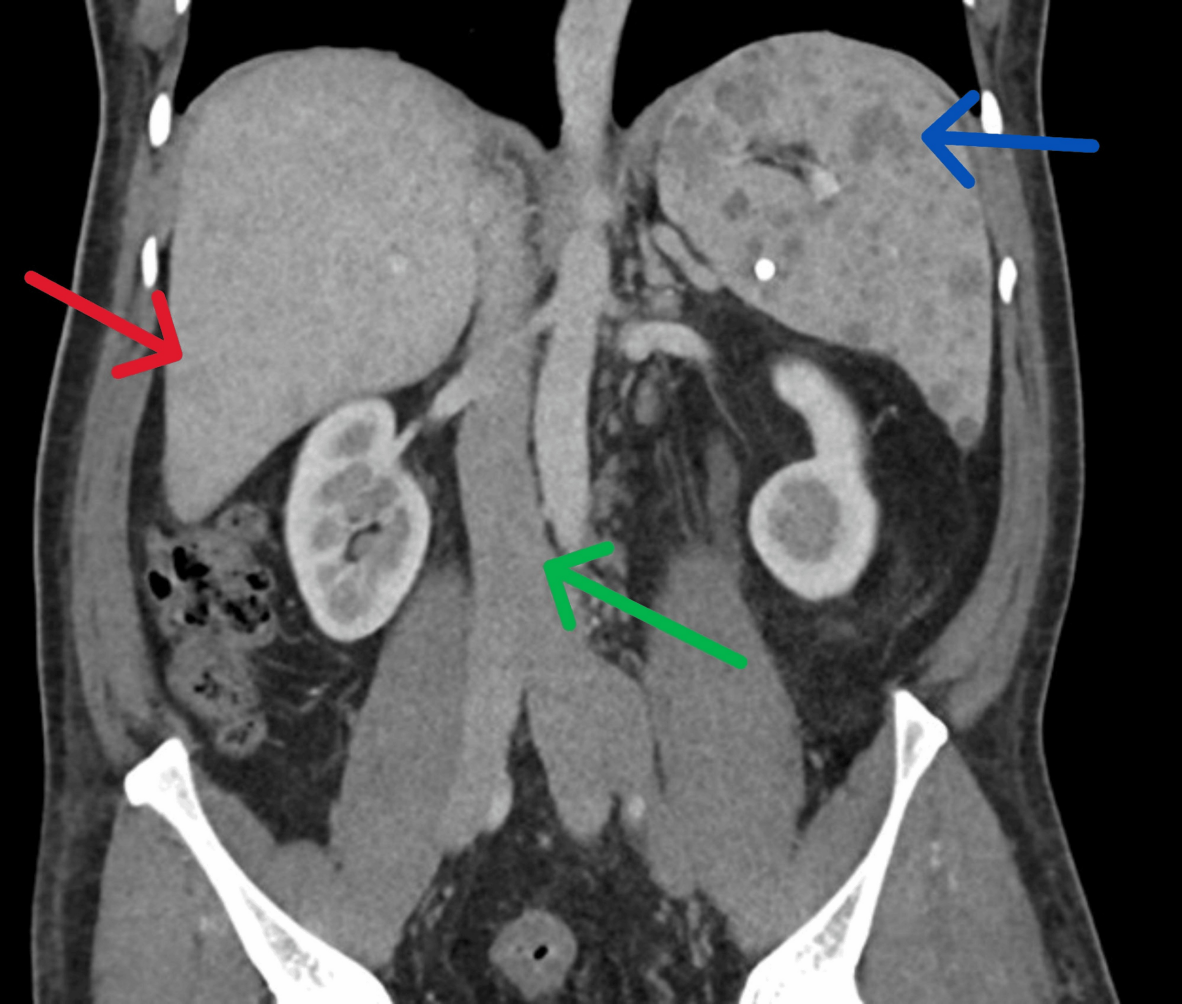
CT abdomen (coronal view) showing innumerable hemangiomas in the liver (red arrow) and spleen (blue arrow), along with an enlarged IVC (green arrow). IVC: inferior vena cava

Further advanced investigation, including computed tomography, revealed mild hepatosplenomegaly with innumerable hemangiomas, rectosigmoid visceral malformation, enlarged inferior vena cava (IVC), congested peritoneal vessels, and mild cardiomegaly, findings consistent with KTS (Figure 4).

**Figure 5:**
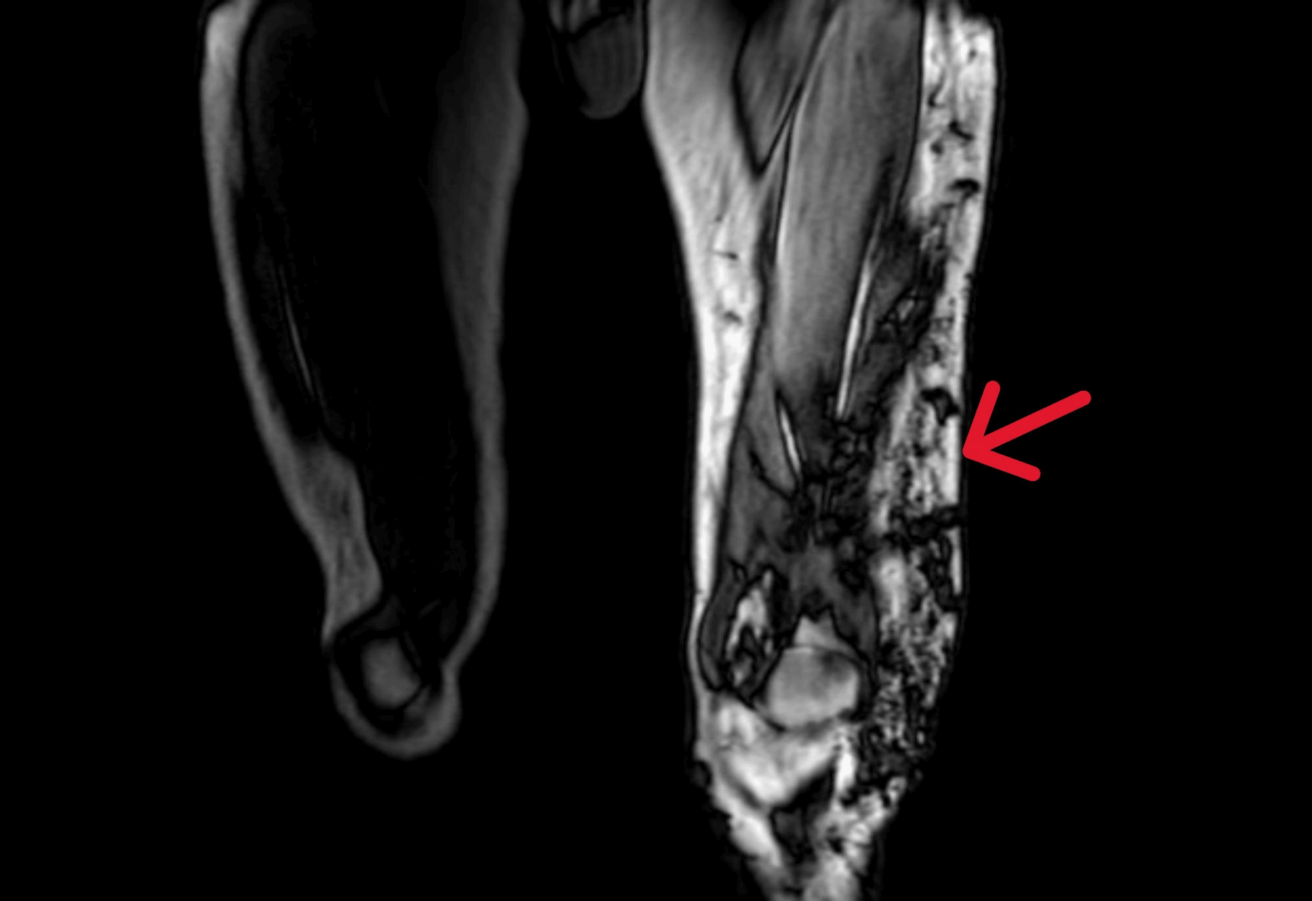
MRI of the left lower limb revealing numerous superficial and deep varicose veins with further superficial draining varicosities. There is diffuse left lower limb enlargement with dilated lymphatics.

Magnetic resonance angiography (MRA) of the left lower limb revealed an extensive slow flow/veno-lymphatic vascular malformation involving the entire length of the left lower limb with limb enlargement and associated edema consistent with KTS (Figure 5).

The patient was diagnosed with KTS based on his medical history, clinical examination, and radiological findings. After his hemoglobin was corrected to above the target level, he was discharged in good condition and referred to the outpatient clinic for follow-up, including colorectal surgery.

## Discussion

KTS is a rare congenital disorder characterized by capillary malformations (like port-wine stains), venous malformations (including varicose veins), and limb overgrowth, which may also involve lymphatic malformations [1-4]. The condition is typically sporadic, although rare familial cases have been documented [5]. KTS is primarily caused by postzygotic somatic mutations in the *PIK3CA* gene, leading to abnormal cell growth and vascular development [7,8].

KTS most often affects the lower limbs but can involve the upper limbs and trunk. Diagnosis is usually based on clinical findings such as skin discoloration and limb enlargement without requiring genetic testing or imaging in straightforward cases. However, imaging may be necessary to assess complications such as venous thrombosis or abnormalities in the IVC. Patients can present with a range of symptoms, from mild overgrowth to significant limb enlargement and varicosities, which often develop early in life. In severe cases, KTS may also affect internal organs such as the liver, spleen, and bladder [11,12].

Venous malformations in KTS include dilated, tortuous veins often visible in the thigh and leg. These veins may become more apparent in childhood, and deeper venous abnormalities may require imaging for detection [13]. Lymphatic malformations in KTS can lead to chronic leakage, infections, and lymphedema, which may contribute to the overgrowth of the affected limb [14]. Overgrowth typically involves soft tissue and bone and can result in limb length discrepancies, which may lead to functional issues such as scoliosis or impaired gait.

Complications associated with KTS include clotting disorders, bleeding (often due to venous malformations), limb length discrepancy, chronic lymphedema, venous insufficiency, and recurrent cellulitis. Pain is also a frequent and debilitating symptom stemming from various factors such as venous insufficiency, thrombophlebitis, and nerve involvement. Pathological features of KTS include capillary and venous malformations, lymphatic anomalies, and secondary complications such as thrombosis and infection [15,16].

The diagnosis of KTS is typically based on clinical features such as vascular stains, venous varicosities, and limb overgrowth, with or without lymphatic malformations. Imaging and lab tests play a key role in confirming the diagnosis. Initial imaging often includes ultrasound and MRI, both helpful in assessing vascular and lymphatic anomalies. Ultrasound, especially Doppler, helps detect venous abnormalities and thrombosis. MRI, with or without contrast, is preferred for its accuracy in defining the extent of vascular and soft tissue abnormalities. Other imaging techniques, such as venography and plain radiographs, can be used for specific evaluations, such as venous anatomy and bone abnormalities [17,18]. In addition, laboratory tests, including blood counts and coagulation panels, help assess the risk of clotting disorders common in KTS patients. While biopsy is usually not required for diagnosis, molecular testing can be valuable, particularly for identifying genetic mutations like *PIK3CA* [19].

Management of KTS involves a multidisciplinary approach, focusing on supportive care and targeted treatments. Compression therapy, physical activity, and regular monitoring are important for managing edema. Some patients may require surgical or medical interventions for complications such as limb overgrowth or chronic venous insufficiency. Recently, targeted therapies such as *PIK3CA* inhibitors (e.g., alpelisib) have emerged as treatment options for more severe cases [11]. Additionally, sirolimus, an mTOR inhibitor, has shown promise in reducing vascular malformations and improving symptoms [20,21]. Pain management, infection control, and prevention of thrombosis are also critical aspects of care.

## Conclusions

This case of KTS highlights a rare complication of acute gastrointestinal bleeding leading to severe anemia. It emphasizes the need for vigilant monitoring of gastrointestinal issues in KTS patients. While KTS generally has a slow progression, early diagnosis and multidisciplinary care are crucial for preventing complications and improving quality of life. Diagnosis is based on identifying at least two key features: varicosities, vascular malformations, and tissue or bone hypertrophy. Management focuses on symptom control and regular follow-up.
